# Protein misfolding disorders and macroautophagy

**DOI:** 10.1016/j.ceb.2010.10.010

**Published:** 2011-04

**Authors:** Fiona M Menzies, Kevin Moreau, David C Rubinsztein

**Affiliations:** Department of Medical Genetics, Cambridge Institute for Medical Research, University of Cambridge, Cambridge, CB 0XY, UK

## Abstract

A large group of diseases, termed protein misfolding disorders, share the common feature of the accumulation of misfolded proteins. The possibility of a common mechanism underlying either the pathogenesis or therapy for these diseases is appealing. Thus, there is great interest in the role of protein degradation via autophagy in such conditions where the protein is found in the cytoplasm. Here we review the growing evidence supporting a role for autophagic dysregulation as a contributing factor to protein accumulation and cellular toxicity in certain protein misfolding disorders and discuss the available evidence that upregulation of autophagy may be a valuable therapeutic strategy.

## Introduction

Protein misfolding disorders or proteinopathies appear, at the clinical level, to be a diverse group of disorders encompassing many diseases, from late-onset neurodegenerative disorders through to forms of heart failure. These conditions are unified by the common feature of accumulation of misfolded proteins. The specific protein, cell type and cellular localisation of these accumulations vary between the diseases. For example, Parkinson's disease is characterised by the presence of cytoplasmic aggregates of α-synuclein, whereas in the polyglutamine expansion disorders, aggregates are seen predominantly within the nucleus in spinocerebellar ataxia type 1, or in the cytoplasm in adult-onset Huntington's disease. In Alzheimer's disease (AD), both intracellular tau aggregates and extracellular amyloid-β (Aβ) aggregates are seen.

**Figure 1:**
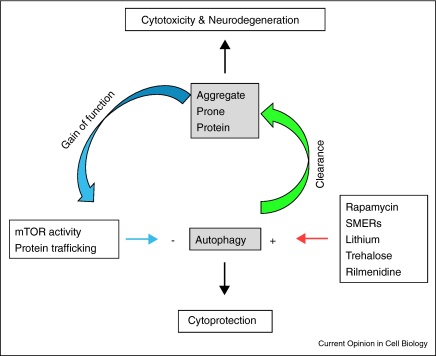
A model for autophagy regulation in proteinopathies. An interconnected system exists in which autophagy is able to control the levels of intracytoplasmic aggregate-prone proteins (green arrow). However, these proteins are also able to control levels of autophagy (blue arrows). Additionally levels of autophagy are also able to be regulated using drugs (red arrow). The balance between these factors will alter cell survival.

Macroautophagy, which we will refer to as autophagy, is an intracellular process in which cytoplasmic materials are engulfed by double membrane structures, which form autophagosomes. The autophagosomes first fuse with endosomes to form hybrid organelles called amphisomes that later fuse with lysosomes, where the entrapped cytosolic contents are degraded. The mechanisms of autophagy are described elsewhere in this review issue. The process of macroautophagy has been proposed to be important in protein misfolding disorders, both as a contributing factor, through inhibition of the process, and a potential therapeutic strategy, through its upregulation (Figure 1). Here we will discuss the evidence for both these possibilities, concentrating on the neurodegenerative proteinopathies in which misfolded protein accumulation is seen in the central nervous system.

## Inhibition of macroautophagy as a contributing factor in proteinopathies

Macroautophagy is induced following various stimuli, the most studied of which is starvation, where upregulation of autophagy acts to provide vital cellular nutrients. In the brain there was thought to be little induction of autophagy following starvation [1], although this view has been challenged by a recent study suggesting profound autophagy upregulation after starvation [2]. Clarification of this issue may require refinement of methods to assess autophagic flux in the brain. In post-mitotic cells, such as neurons, turnover of proteins and organelles is particularly important for cellular quality control, and basal (or constitutive) autophagy appears to be vital to maintain this. Complete knockout of the essential autophagy genes Atg5 or Atg7 in mice causes lethality soon after birth [3,4]. However, selective knockout of these genes in neuronal cells results in a phenotype closely resembling those seen in neurodegenerative diseases, as well as protein aggregation without the expression of a disease-causing protein [5,6].

There is also accumulating evidence for the fact that aggregating, misfolded proteins may have an impact on autophagic function, suggesting that this could be a secondary pathological mechanism in many diseases. This aspect will be discussed below.

### Huntington's disease

Huntington's disease (HD) is a hereditary neurodegenerative disease resulting from an expansion of the polyglutamine region of the ubiquitously expressed protein huntingtin (htt) [7]. This mutant protein accumulates inside cells, forming toxic oligomeric species and aggregates. Immunohistochemistry and electron microscopy approaches, either in HD patients or experimental models, have suggested alterations in the autophagic pathway. The earliest evidence comes from observations of increased numbers of autophagic vacuoles across a range of HD models and in patients [8–13]. While it is not clear if this is due to enhanced autophagosome formation or decreased clearance of the vesicles, mTOR is inactivated by cells with mutant huntingtin inclusions, as it is sequestered into aggregates, and this would be compatible with autophagy upregulation [14].

More recently, it has been reported that the autophagic turnover of cytoplasmic components is partially impaired in cells expressing mutant huntingtin (Table 1) [15^•^]. While autophagosomes are able to form and fuse with lysosomes, the authors report that expression of mutant huntingtin results in inefficient cargo loading, although mutant huntingtin is efficiently delivered to the autophagosomes. This preferentially affects organelle sequestration, in particular that of lipid droplets, which are seen to be increased in Huntington's disease, and mitochondria, dysfunction in which have been widely reported in Huntington's disease [16].

Further evidence for a contribution of autophagy to pathogenesis in HD comes from recent genetic studies, which suggest that the V471A Atg7 polymorphism is associated with earlier age of onset [17]. Whether or not this polymorphism has any effect on autophagic activity has yet to be established.

### Alzheimer's disease

The (Aβ) peptide plaques, which characterise AD, are derived from proteolysis of amyloid precursor protein (APP). Mutations in APP and presenilin (PS1), a protein involved in APP to Aβ proteolysis, cause rare autosomal dominant forms of familial Alzheimer disease (FAD) [18–20]. Sporadic AD, which is far more prevalent, presents the same clinical and pathological characteristics, which suggest that factors affecting the APP to Aβ pathway play a significant role in this form of the disease [21].

A prominent feature of AD is the accumulation of autophagosomes, many containing amyloid-β peptide, in massive numbers within affected neurons [22], probably reflecting defective autophagosome clearance [23]. Changes in the autophagic pathway have been linked to AD through diverse mechanisms, however, it is not clear if autophagosome formation is increased or decreased. A decrease in formation is supported by the reduction in levels of the autophagy protein Beclin-1 [24^••^] or by inhibition of Beclin-1 activity by the HSV1 (Herpes Simplex Virus Type 1) viral protein ICP34.5 (Table 1) [25]. An increase in autophagosome formation is supported by data suggesting that Aβ is able to induce autophagy via the generation of reactive oxygen species [26^•^].

Lysosome-related pathology, along with neuronal loss and amyloid deposition, is greatly accentuated in FAD due to mutations of PS1 [27]. PS1 appears to regulate proteolysis during autophagy by targeting the v-ATPase to lysosomes [28^••^]. PS1 in the ER acts as a chaperone to facilitate maturation and targeting of the v-ATPase V0a1 subunit to lysosomes, which is essential for acidification, protease activation, and degradation of autophagic/lysosomal substrates and could account for the accumulation of autophagosomes seen in AD.

### Parkinson's disease

Parkinson's disease (PD) is characterised by the presence of intraneuronal cytoplasmic inclusions known as Lewy bodies, of which α-synuclein is a major constituent [29]. Several mutations have been identified in autosomal recessive forms of PD that provide some insight into the pathogenesis of this disease. Recent studies have linked two such genes, PINK1 and Parkin with mitochondrial clearance and autophagy (Table 1). Parkin has been demonstrated to be recruited to damaged mitochondria and promote their clearance by autophagy [30^•^] in a manner that is dependent on the stabilisation of Pink1 on the mitochondria [31^•^,32^•^,33^•^,34^•^]. This translocation is disrupted by mutations in Pink1 or Parkin seen in familial PD [31^•^,32^•^]. A direct interaction between Pink1 and Beclin-1 has also been demonstrated recently, promoting autophagosome formation [35], further strengthening the link between autophagy and PD.

Mitochondrial dysfunction has also been linked to mutations of DJ-1 (PARK7), another autosomal recessive PD gene [36]. Its loss, which can be rescued by the expression of Pink1 and Parkin, leads to increased susceptibility of neurons to oxidative stress and death. Interestingly, DJ1-deficiency leads to increased autophagic activity [36], probably with the aim of clearing dysfunctional mitochondria, by a mechanism that remains to be established, but could involve ROS production, mTOR signalling or direct interaction with Pink1/Parkin pathway [36].

Autosomal dominant mutations in leucine-rich repeat kinase 2 (LRRK2) are the most common genetic cause of PD [37,38]. LRRK2 loss causes phenotypes that may be due to impairment of the autophagy-lysosomal pathway, like the accumulation of α-synuclein and apoptotic cell death in aged mice [39]. The effect of LRRK2 on autophagy still remains to be elucidated but might involve the formation of multivesicular bodies (MVB) or the inhibition of the UPS system following the accumulation of α-synuclein (Table 1).

Mutations in the α-synuclein gene, including point mutations and multiplications of the entire locus, have been shown to cause autosomal dominant forms of PD, although the mechanism still remains obscure. In yeast, overexpression of α-synuclein perturbs the secretory pathway by inhibiting Rab1 activity [40]. We recently found that α-synuclein overexpression causes autophagy inhibition by inhibiting Rab1a [41^•^].

### Dementia and amyotrophic lateral sclerosis

A group of neurodegenerative proteinopathies, such as motor neuron diseases (MND), are associated with defects in autophagosome trafficking. Disruption of retrograde axonal transport of cargo by overexpression or depletion of dynein complex components in transgenic mice results in the progressive degeneration of motor neurons and the formation of inclusions, mimicking the phenotype in some MND patients [42–44]. As dynein activity is crucial for microtubule-based delivery of autophagosomes to lysosomes, mutations in the dynein machinery impair autophagosome clearance. Indeed, an increase in autophagosome number and LC3-II levels can be observed in mice with dynein mutations [45,46]. Further research will be needed to clarify the relative importance of autophagy dysfunction in MND, particularly in forms not due to primary mutations of the dynein machinery, but where axonal transport deficiencies have been reported.

Another group of diseases that manifest impaired autophagic flux are due to mutations in the ESCRT complex machinery, which has been implicated in neurodegenerative disorders, such as frontotemporal dementia linked to chromosome 3 (FTD3) [47] and amyotrophic lateral sclerosis (ALS) [48,49]. Expression of a deletion mutant of CHMP2B, a subunit of the ESCRT-III complex, in cell and fly models, increased LC3-II levels and caused an accumulation of autophagosomes [50]. Experimental characterisation of CHMP2B indicates that the proper dissociation of the ESCRT-III complex is crucial to both autophagosome maturation and proper fusion of autophagosomes with lysosomes [51].

Further evidence suggesting a perturbation of autophagy may contribute to pathogenesis of ALS and other associated disorders comes from studies of the lipid phosphatase Fig4. Mutations in this gene are responsible for Charcot-Marie-Tooth disease type 4 and a variant has also been described in ALS patients [52]. A decrease in PI(3,5)P_2_ levels mice lacking Fig4 has been reported, and this is associated with alterations in autophagic markers consistent with a decrease in autophagy in these animals [53].

### Lafora disease

Lafora disease (LD) is an autosomal recessive myoclonus epilepsy. Its pathological hallmark is the accumulation of polyglucosan inclusions, called Lafora bodies, in the cytoplasm of cells in many organs. The majority of mutations causing LD occur in two genes: *EPM2A*, which encodes laforin, and *EPM2B*, which codes for malin [54]. It has recently been demonstrated that a deficiency in autophagy may contribute to the accumulation of Lafora bodies [55^•^]. Data obtained in patient cells, laforin knockout mice and in cell culture systems showed that laforin is a positive regulator of autophagy (Table 1). Loss of laforin resulted in an increase in the activity of the negative regulator of autophagy, mTOR. The exact mechanism for the laforin effect on autophagy is still elusive, as specific laforin substrates relevant to autophagy have yet to be identified.

## Protection by autophagy induction in protein misfolding disorders

While progress has been made in understanding the molecular pathology of protein misfolding disorders, exactly how these proteins cause cellular toxicity still remains to be elucidated. However, extensive data suggest that toxicity is mediated primarily via gain-of-function mechanisms (Figure 1). Disease severity appears to correlate with the level of expression of the mutant protein, while loss-of-function mutants often show phenotypes distinct from the disease. Whether it is the aggregated protein, the soluble protein, or an intermediate oligomeric form that confers this toxic gain-of-function is a matter of controversy. However, the large aggregates visible by light microscopy may not be the most toxic species [56]. Regardless of the exact toxic species, the fact these diseases result from a toxic gain-of-function means that the efficiency of the removal of the mutant proteins from the cell is likely to be an important factor in their toxicity.

In general, intracytoplasmic aggregate-prone proteins are good autophagy substrates [57,58], although there are some exceptions to this [59]. Clearance by autophagy has been demonstrated for both wild-type and mutant forms of tau [58], mutant forms of α-synuclein that cause familial Parkinson's disease [60] and a range of polyglutamine-expanded proteins [14,57,58,61]. Interestingly, the non-aggregate-prone species of many of these proteins (e.g. huntingtin and α-synuclein) show a much lower dependency on autophagy for their clearance, compared to their mutant counterparts [60,62–64]. This may have additional benefits in certain neurodegenerative diseases, allowing preferential clearance of the mutant/aggregate-prone protein, without affecting wild-type protein levels.

The importance of autophagy in aggregate-prone protein clearance is probably increased due to the proteasome being unable to degrade oligomeric species, as they cannot be unfolded to enter the narrow barrel of the proteasome [65]. Additionally, in the case of polyglutamine proteins, the proteasome may be unable to cleave within the polyglutamine tract [65,66]. By contrast, autophagosomes are able to engulf oligomeric species. This does not mean that autophagy clears purely aggregated species, as its upregulation results in a decrease in the levels of both soluble and aggregated protein forms [60]. Equally, it is not known if autophagy can clear large aggregates directly or if a decrease in aggregates is seen due to a decrease in the soluble levels of the protein [67,68], although aggregates visible by light microscopy are not membrane-bound, suggesting that earlier and smaller oligomeric structures are the autophagy substrates.

## Therapeutic implications for upregulation of autophagy

Evidence for the importance of autophagy in the degradation of mutant, misfolded proteins has wide therapeutic implications in the treatment of proteinopathies. We initially demonstrated that upregulation of autophagy, using the mTOR inhibitor rapamycin or its water-soluble ester CCI-779, reduced levels of soluble and aggregated mutant huntingtin and was protective in cell, *Drosophila* and mouse models of Huntington's disease [14]. Further to this it has been demonstrated that genetic inhibition of mTOR, by overexpression of TSC1 and TSC2, conferred neuroprotective effects in a *Drosophila* model of Huntington's disease [69]. Indeed, the therapeutic scope for upregulation of autophagy by mTOR inhibitors extends beyond Huntington's disease, as CCI-779 treatment also demonstrated protective effects in a mouse model of spinocerebellar type 3 [70].

As the most common neurodegenerative disease, possible treatments for AD are of great interest, and there is now evidence that a rapamycin treatment may be beneficial. Aβ levels were decreased and cognitive defects were prevented by rapamycin treatment in two different AD mouse models [71^•^,72^•^]. Further evidence for a potential protective role of autophagy upregulation in AD comes from genetic studies. Overexpression of Beclin-1, an autophagy regulating gene, in AD mice models reduced intracellular accumulation of Aβ and extracellular deposition of Aβ plaques [24^••^]. This strategy of autophagy upregulation has also proved beneficial in a mouse model of Parkinson's disease overexpressing α-synuclein [73].

While mTOR inhibitors show some potential as therapeutic agents, mTOR has multiple roles in cellular homeostasis, and thus mTOR inhibitors are likely to have side-effects due to modulations in processes other than autophagy. It has therefore become a priority to identify other drugs that induce autophagy via mTOR-independent mechanisms. Screening of United States Food and Drug Administration-approved drugs with this aim in mind, identified a number of drugs that were able to upregulate autophagy and protect against toxicity in cell, *Drosophila* and zebrafish models of Huntington's disease [74]. One of these drugs, rilmenidine, a centrally acting anti-hypertensive, has subsequently been shown to be protective in a mouse model of Huntington's disease [75^•^].

Lithium induces autophagy by lowering intracellular inositol or inositol 1,4,5-trisphosphate (IP3) levels in an mTOR-independent manner [76], and it has been shown to delay disease progression in a mouse model of ALS overexpressing a mutant form of SOD1 and also in a small trial with ALS patients [77]. Cellular studies have previously demonstrated the requirement of autophagy for the clearance of SOD1 [78], and more recently for TDP-43, another aggregate-prone protein, mutations in which cause ALS [79,80] suggesting that there may be therapeutic potential for the upregulation of autophagy in ALS. Further support for this comes from evidence that XBP-1 deficiency protects against neurodegeneration in mice overexpressing mutant SOD1 [81]. XBP-1 is a transcription factor required for induction of the unfolded protein response (UPR), and it was therefore predicted that its loss would increase the toxicity of mutant SOD in a mouse model. However, deficiency of XBP-1 markedly attenuated development of disease signs, as it resulted in an increase in autophagy and decreased levels of SOD1 aggregation [81].

Autophagy can also be upregulated in an mTOR-independent manner by the disaccharide trehalose [64]. Treatment of a mouse model of tauopathy with parkinsonism, in which mutant tau is overexpressed along with a parkin deletion, resulted in a decrease in the levels of phosphorylated tau and protection against loss of dopaminergic neurons [82^•^]. Trehalose has previously been shown to have neuroprotective effects, which were attributed to its activity as a chemical chaperone (see [83] for review). However, in this mouse model, an upregulation of autophagy was seen, confirming the potential for dual mechanisms of protection by trehalose.

## Future perspectives

For many diseases, the upregulation of autophagy is a promising therapeutic target. Combining knowledge of the potential mechanisms of autophagy compromise in neurodegenerative proteinopathies with knowledge of the range of signalling pathways and drugs available to control autophagy may make the development of therapeutics based on this process possible.

## References and recommended reading

Papers of particular interest, published within the period of review, have been highlighted as:

• of special interest
•• of outstanding interest

## Figures and Tables

**Table 1:** Regulation of autophagy in proteinopathies

Disease	Mutant protein	Autophagy activity	Mechanism
Alzheimer	–	Inhibition (AV formation)	Beclin-1 targeting by HSV protein ICP34.5
	–	Inhibition (AV formation)	Beclin-1 inhibition
	PS1	Inhibition (AV maturation)	Lysosome acidification (v-ATPase targeting)
	–	Induction (AV formation)	ROS production
Parkinson	α-Synuclein	Inhibition (AV formation)	Rab1 activity; Atg9 localisation
	LRRK2	Inhibition (AV maturation)	MVB formation; UPS impairment
	PINK1	Inhibition (AV formation and mitophagy)	PINK1/Beclin-1 interaction; mitochondria targeting
	Parkin	Inhibition (mitophagy)	Mitochondria targeting
	DJ-1	Induction (AV formation)	ROS production; mTOR
Huntington	Huntingtin	Inhibition (selectivity)	Cargo recognition
Lafora	Laforin	Inhibition (AV formation)	mTOR activation
	Malin	Unknown	
ALS	Dynein	Inhibition (AV maturation)	Autophagosome transport to lysosome
	Dynactin	Unknown	
	ESCRT-III	Inhibition (AV maturation)	Autophagosome/lysosome fusion
	Fig4	Inhibition	Decreased PI(3,5)P_2_ levels
FTD3	ESCRT-III	Inhibition (AV maturation)	Autophagosome/lysosome fusion
